# Clinical implications of real-time optic nerve sheath diameter assessment via critical care ultrasound in intracranial hypertension

**DOI:** 10.3389/fneur.2025.1488482

**Published:** 2025-02-13

**Authors:** Nan Zhang, Meng Liang, Tenghao Shao, Ning Li, Kuo Wang, Shuo Sun, Tao Sun

**Affiliations:** ^1^Department of Critical Care Medicine, Affiliated Hospital of Hebei University, Baoding, Hebei, China; ^2^Department of Hepatobiliary Surgery, Affiliated Hospital of Hebei University, Baoding, Hebei, China; ^3^Department of Neurosurgery, Affiliated Hospital of Hebei University, Baoding, Hebei, China

**Keywords:** critical care ultrasound, intracranial hypertension, optic nerve sheath width, real-time monitoring, ultrasound measurement

## Abstract

**Objectives:**

This study aims to assess the clinical value of dynamic monitoring of optic nerve sheath diameter using critical care ultrasound in the management of patients with intracranial hypertension.

**Methods:**

A total of 130 patients with craniocerebral injuries, treated at the Department of Critical Care Medicine of the Affiliated Hospital of Hebei University from January 2021 to November 2022, were selected and randomly assigned to either the control group (65 patients) or the observation group (65 patients). Patients in both groups were monitored based on clinical symptoms, cranial CT findings, and optic nerve sheath diameter (ONSD). The control group received standard osmotic therapy to manage intracranial pressure (ICP), while the observation group was guided accordingly. Comparative analyses were conducted on Acute Physiology and Chronic Health Evaluation II (APACHE II) scores, Glasgow Coma Scale (GCS) scores, duration of ICU stay, and mechanical ventilation time between the two groups.

**Results:**

On the 28th day, the APACHE II scores of patients with craniocerebral injuries in both groups were significantly lower compared to admission scores, while GCS scores were higher (*P* < 0.05). Compared to conventional management, the observation group showed a 15% reduction in APACHE II scores, a 20% decrease in ICU stay duration, and a 25% reduction in mechanical ventilation time by day 28 post-admission. The observation group also showed a higher proportion of patients with favorable prognoses and a significant reduction in severe disability and vegetative survival rates (*P* < 0.05).

**Conclusion:**

Dynamic monitoring of ONSD using bedside critical care ultrasound has proven effective in guiding osmotic therapy for patients with intracranial hypertension. This approach significantly reduces ICP, offers a reliable basis to opt for subsequent treatments, and effectively lowers the rate of disability while improving patient prognosis.

## 1 Introduction

Intracranial pressure (ICP) refers to the pressure exerted by the contents within the cranial cavity against its walls. Elevated ICP, known as intracranial hypertension, can lead to brain tissue compression, resulting in ischemia, hypoxia, coma, brain dysfunction, or even fatality. This condition is a common cause of fatality in clinical settings (1). Predicting ICP elevation is challenging, and traditional invasive ICP monitoring techniques, such as lumbar puncture and craniotomy, require advanced physician skills and carry risks of complications including intracranial infection and hemorrhage (2). Consequently, there is significant clinical value in identifying a simple, rapid, and non-invasive method for assessing ICP changes. Recent literature indicates that ultrasound measurement of optic nerve sheath diameter (ONSD) can effectively assess ICP levels due to its simplicity, non-invasiveness, and ability to provide real-time, dynamic data (3). While current research has primarily examined the correlation between invasive ICP monitoring and ONSD measurements via ultrasound, studies on the impact of dynamic ONSD monitoring for evaluating ICP reduction through dehydration in patients with craniocerebral injuries are limited. The aim of this study is to explore the effectiveness of ONSD measurement by critical care bedside ultrasound in guiding ICP reduction through dehydration in such patients.

## 2 Data and methods

### 2.1 Clinical data

A total of 188 patients with craniocerebral injuries, admitted to the intensive care unit of our hospital between January 2021 and November 2022, were included in the study. The cohort comprised 116 males and 72 females, aged 18 to 88 years, with a mean age of 56 years. Diagnoses among these patients primarily included craniocerebral trauma, intracranial hemorrhage, subarachnoid hemorrhage, and extensive cerebral infarction. Exclusion criteria encompassed patients with glaucoma, severe ocular trauma, optic neuritis, optic nerve tumors, or other ocular diseases, as well as those with a hospital stay of < 24 h. Ultimately, 130 patients met the inclusion criteria.

### 2.2 Treatment methods

Both patient groups were administered a standard dehydration regimen, which typically included mannitol at 0.5 g/kg every 8 h or hypertonic saline. The frequency of osmotic therapy could be adjusted based on clinical conditions, and furosemide and protein could be added as needed. Analgesia and sedation were managed strictly according to the Critical Care Pain Observation Tool (COPT) and the Richmond Agitation-Sedation Scale (RASS). Vital signs, blood routine, electrolytes, and other relevant indicators were continuously monitored. Fluid balance was carefully maintained, and active nerve nutritional therapy was provided.

**Figure 1:**
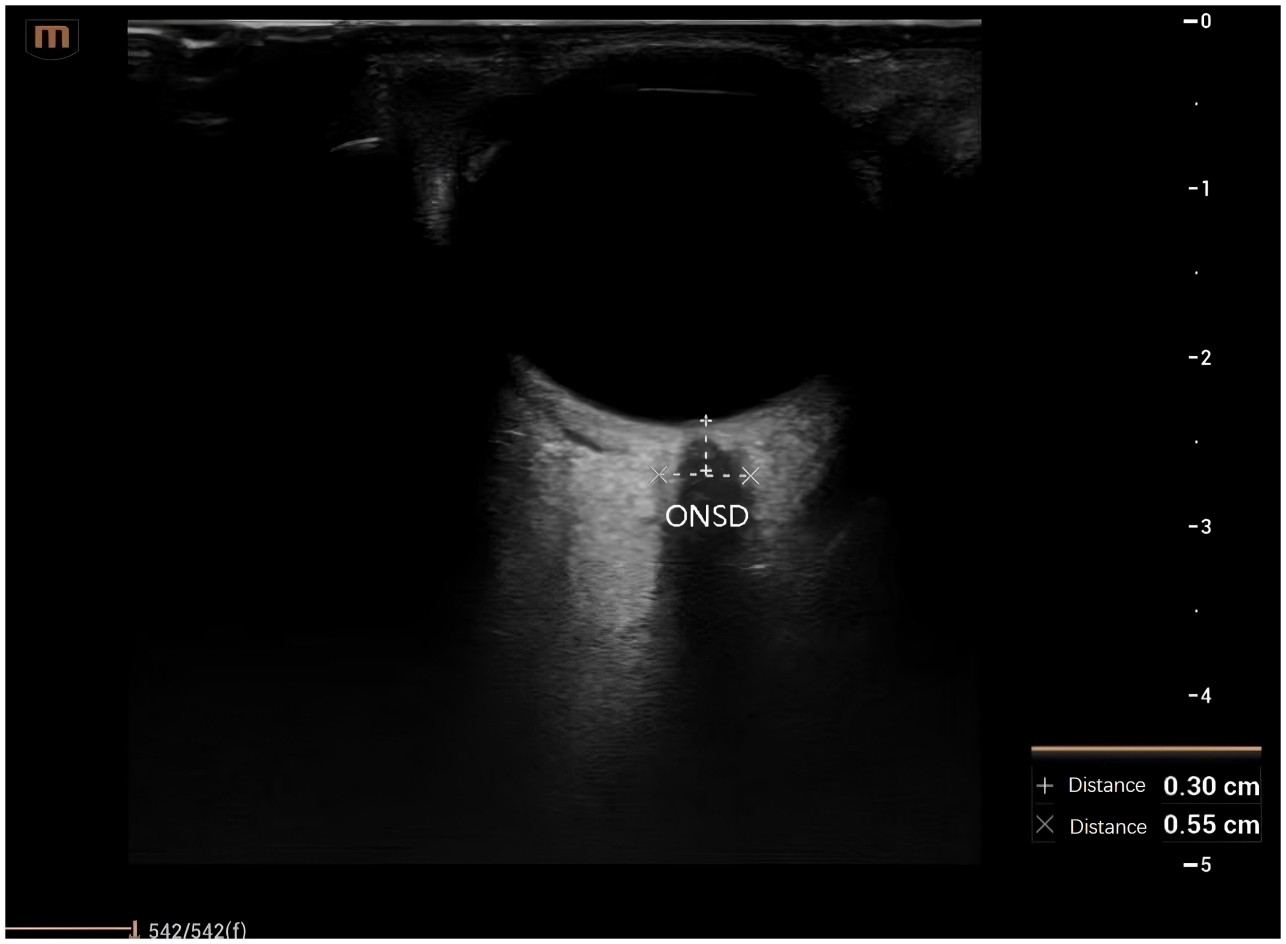
Ultrasound assessment and measurement of the optic nerve. ONSD, optic nerve sheath diameter.

In the test group, while patients received standard treatment, qualified physicians from the Department of Critical Care Medicine performed ONSD measurements using Sonosite ultrasound equipment (model: M-Turbo). Measurement procedure: Patients were placed in the supine position with eyes gently closed. A transparent film was placed over the eyelids, and a high-frequency probe, coated with an appropriate coupling agent, was held like a pen and positioned at the center of the eyelid for a transverse examination. This procedure visualized the optic nerve in its long axis, with the optic nerve sheath appearing as a strip-shaped hypoechoic structure extending from the optic nerve head into the cranium. ONSD was measured 3 mm behind the optic nerve head. Each eye was measured three times, and the average value was calculated. ONSD measurements were taken 30 min prior to the daily administration of mannitol or hypertonic saline. An ONSD ≥ 4.8 mm indicated elevated ICP, prompting immediate adjustment of the osmotic therapy to maintain the ONSD below the target value of < 4.8 mm (Figure 1).

In the control group, ICP was monitored using conventional methods, with ICP assessment based on clinical symptoms or brain CT imaging. Patients underwent brain CT scans at 12, 24, and 72 h post-surgery. Any clinical changes, such as anisocoria, deterioration in consciousness, headache, or alterations in cranial imaging (e.g., significant brain tissue swelling, midline shift, brainstem injury, or cisterna ambiens compression), indicated elevated ICP. In such cases, osmotic therapy regimen of the patient was adjusted empirically.

### 2.3 Observation indicators

(1) APACHE II scores and Glasgow Coma Scale (GCS) scores of patients upon admission and 28 days after admission.

(2) Prognostic indicators for patients include the Glasgow Outcome Scale (GOS) and the treatment effectiveness rate.

(3) Additional evaluation indicators include the duration of ICU stay and the length of mechanical ventilation.

### 2.4 Criteria for determining patients' prognosis and efficacy

In this study, GOS was utilized to assess the prognosis of patients with craniocerebral injuries (4). The scale is categorized as follows: (1) Good (5 points) indicates that the patient has fully recovered and can return to normal work and study activities, with follow-up CT scans showing no abnormalities; (2) Moderate disability (4 points) denotes that the patient experiences some neurological symptoms but remains self-sufficient, with follow-up CT scans appearing largely normal; (3) Severe disability (3 points) implies that the patient maintains a clear conscious state but requires assistance from others, with follow-up CT scans showing improvement compared to previous results; (4) Vegetative survival (2 points) represents a state of coma or persistent vegetative state, with no improvement or deterioration in condition; (5) Fatality (1 point). A GOS score of ≤ 3 indicates an unfavorable prognosis, while a score of >3 reflects a favorable prognosis.

### 2.5 Statistical processing

Data analysis was conducted using SPSS version 23.0. Measurement data, which met the criteria for normal distribution and homogeneity of variance, were expressed as mean ± standard deviation (). Comparisons between two groups were performed using paired *t*-tests or independent samples *t*-tests. Categorical data were presented as frequencies (n) or percentages (%), and comparisons between groups were made using the χ^2^ test. A *P* < 0.05 was considered indicative of statistical significance.

## 3 Results

### 3.1 Comparison of APACHE II score and GCS score between the two groups on admission and 28d after admission

At admission, there were no statistical differences in APACHE II scores and GCS scores between the two groups of patients with intracranial hypertension (*P* > 0.05). Compared to admission values, both groups exhibited significant reductions in APACHE II scores and significant increases in GCS scores, with these changes being statistically significant (*P* < 0.05). Twenty-eight days after admission, the observation group showed a more pronounced decrease in APACHE II scores and a greater increase in GCS scores compared to the control group, with these differences also being statistically significant (*P* < 0.05; see Table 1).

**Table 1:** Comparison of APACHE II scores and GCS scores between the two patient groups at admission and 28 days after admission.

	**Control group (*n* = 65)**	**Observation group (*n* = 65)**	***P*-value**
**APACHE II score**			
Admission	21.87 ± 3.60	22.13 ± 3.86	>0.001 (0.864)
28 d after admission	15.55 ± 2.18	10.72 ± 1.63	< 0.001 (4.33e-17)
**GCS score**			
Admission	8.49 ± 1.61	8.31 ± 1.45	>0.001 (0.378)
28 d after admission	12.03 ± 2.76	13.91 ± 2.27	< 0.001 (4.19e-11)

APACHE II, Acute Physiology and Chronic Health Evaluation II; GCS, Glasgow Coma Scale.

### 3.2 Comparison of ICU stay time and mechanical ventilation time between the two groups

Compared to the control group, the observation group experienced a significantly shorter duration of ICU stay and mechanical ventilation time, with these differences being statistically significant (see Table 2).

**Table 2:** Comparison of ICU length of stay and duration of mechanical ventilation between the two patient groups.

	**Control group (*n* = 65)**	**Observation group (*n* = 65)**	***P*-value**
ICU stay time	17.38 ± 3.19	12.01 ± 2.38	< 0.05
Mechanical ventilation time	8.95 ± 2.19	6.38 ± 1.19	< 0.05

ICU, Intensive Care Unit.

### 3.3 Comparison of GOS between the two groups

In comparison to the control group, the observation group had a significantly higher proportion of patients with a favorable prognosis, with the difference being statistically significant (*P* < 0.05). Additionally, the proportions of severe disability and vegetative survival were significantly lower in the observation group, with these differences also being statistically significant (*P* < 0.05). No statistical differences were observed between the two groups regarding moderate disability, mortality, and overall treatment effectiveness (see Table 3).

**Table 3:** Comparison of GOS scores between the two patient groups.

**Treatment effectiveness**	**Control group (*n* = 65)**	**Observation group (*n* = 65)**	***P*-value**
Good (*n*, %)	19 (29.2)	32 (49.2)	0.018
Moderate disability (*n*, %)	14 (21.5)	23 (35.4)	0.063
Severe disability (*n*, %)	12 (18.5)	3 (4.6)	0.004
Vegetative survival (*n*, %)	13 (20)	4 (6.2)	0.006
Death (*n*, %)	7 (10.8)	2 (3.1)	0.059
Total effective rate (*n*, %)	45 (69.2)	58 (89.2)	0.094

GOS, Glasgow outcome scale.

## 4 Discussion

Elevated ICP can alter cerebral perfusion pressure and cerebral blood flow, leading to cerebral ischemia, hypoxia, and potential brain herniation, which significantly impacts prognosis of patient. Effective dehydration to reduce ICP can improve patient conditions and enhance survival rates (5). Consequently, employing an effective dehydration regimen is crucial. The aim of this study was to compare the effectiveness of dehydration regimens guided by clinical manifestations or intracranial imaging versus those guided by ONSD measurements in patients with intracranial hypertension, to identify an optimal dehydration strategy for managing this condition (6).

ICP is challenging to predict and can progress rapidly. In current clinical practice, ICP is primarily assessed using both invasive and non-invasive methods, with invasive monitoring being considered the gold standard for diagnosing elevated ICP (7). Invasive techniques include lumbar puncture, ventricular manometry, and similar procedures. Non-invasive methods encompass cranial CT, MRI, evoked potentials, and transcranial Doppler ultrasonography. However, invasive tests are complex and carry risks of complications such as infection, hemorrhage, and brain damage, which may exacerbate the condition of patient (8). Non-invasive clinical methods also have limitations, including the need for patient transfer, high subjectivity, or lack of available equipment. Bedside ultrasound, as a non-invasive diagnostic tool, offers significant advantages due to its ability to provide continuous monitoring and better diagnostic value (9).

The subarachnoid space of the optic nerve is in continuous communication with the cerebrospinal fluid (CSF) in the cranial subarachnoid space, and the optic nerve sheath is an extension of the dura mater. A transverse subarachnoid space allows for the gradual drainage of CSF (10). When ICP rises, CSF leakage from the arachnoid can lead to persistent dilation of the interstitial space surrounding the optic nerve. This dilation reduces blood flow, impairs circulation, causes venous stasis, and results in an increase in ONSD (11, 12).

Research has shown that assessing ONSD can offer a more accurate evaluation of intracranial hypertension (13). Bedside measurement of ONSD using critical care ultrasound has proven to be highly effective and feasible for diagnosing intracranial hypertension (14). Furthermore, patients with intracranial hypertension admitted to the ICU typically present with higher APACHE II scores and lower GCS scores. Following dehydration aimed at reducing ICP, restoring consciousness, and providing cranial nerve support, both APACHE II scores and GCS scores improved, making these metrics valuable for evaluating the severity of intracranial hypertension. In this study, APACHE II scores in both the observation and control groups were significantly lower, and GCS scores were significantly higher 28 days after admission compared to initial scores, indicating the effectiveness of both dehydration regimens based on conventional monitoring and ONSD monitoring. However, the APACHE II score in the observation group was notably lower and the GCS score notably higher compared to the control group after 28 days, suggesting that the ONSD-based regimen was more effective. In clinical practice, conventional dehydration regimens based on clinical symptoms or CT imaging lack real-time monitoring and exhibit greater subjectivity. In contrast, ONSD examination enables real-time dynamic monitoring at the bedside, allowing for timely and accurate adjustments to dehydration treatment while minimizing external interference, thereby enhancing therapeutic outcomes.

The GOS score is a key indicator for assessing the prognosis of patients with craniocerebral injuries. According to this study, the proportion of patients with a favorable prognosis in the observation group was higher compared to the control group. Additionally, the observation group had a significantly reduced proportion of severe disability and vegetative survival. The ICU stay duration and mechanical ventilation time were also significantly shorter in the observation group than in the control group. These findings suggest that modifying the dehydration regimen based on ONSD measurements can enhance treatment efficacy and improve prognosis of patient.

This study further validates the effectiveness and clinical utility of critical care ultrasound for measuring ONSD in diagnosing and managing intracranial hypertension. However, there are several limitations to consider. First, no statistically significant differences in fatality rates and overall treatment efficacy between the two patient groups were observed in this study. This lack of difference may be attributed to the relatively small sample size, suggesting that further research with a larger cohort is needed to address this issue. Second, the ultrasound measurement of ONSD involves a degree of subjectivity. Despite the operators being blinded to intracranial pressure values and adhering to standardized measurement procedures, variability in the interpretation of high-density and low-density shadows of the optic nerve sheath among different operators remains a potential source of error. This subjectivity may impact the broader clinical adoption of ultrasound-based ONSD monitoring (15–17). To address the limitations of B-scan ultrasound, the Standardized A-scan ultrasound technique has been proposed. This technique provides a more objective and reproducible method for measuring ONSD by analyzing the amplitude of the reflected sound waves. Studies have shown that A-scan ultrasound can reduce inter-operator variability and improve the accuracy of ONSD measurements. Future research should aim to validate the use of A-scan ultrasound in clinical settings and compare its performance with B-scan ultrasound.

## 5 Conclusion

Dynamic monitoring of ONSD using bedside critical care ultrasound has proven to be an effective tool in guiding osmotic therapy for patients with intracranial hypertension. This approach significantly reduces ICP and provides a reliable basis for subsequent treatment decisions, potentially lowering the rate of disability and improving patient prognosis.

## Data Availability

The raw data supporting the conclusions of this article will be made available by the authors, without undue reservation.
